# Clinical Research in Prehospital Care: Current and Future Challenges

**DOI:** 10.3390/clinpract13050114

**Published:** 2023-10-23

**Authors:** Jonathan Cimino, Claude Braun

**Affiliations:** 1 Clinical Research Unit, Fondation Hôpitaux Robert Schuman, 44 Rue d’Anvers, 1130 Luxembourg, Luxembourg; 2 Hôpitaux Robert Schuman, 9 Rue Edward Steichen, 2540 Luxembourg, Luxembourg

**Keywords:** prehospital care, clinical research, emergency medical services, research methodology and infrastructure

## Abstract

Prehospital care plays a critical role in improving patient outcomes, particularly in cases of time-sensitive emergencies such as trauma, cardiac failure, stroke, bleeding, breathing difficulties, systemic infections, etc. In recent years, there has been a growing interest in clinical research in prehospital care, and several challenges and opportunities have emerged. There is an urgent need to adapt clinical research methodology to a context of prehospital care. At the same time, there are many barriers in prehospital research due to the complex context, posing unique challenges for research, development, and evaluation. Among these, this review allows the highlighting of limited resources and infrastructure, ethical and regulatory considerations, time constraints, privacy, safety concerns, data collection and analysis, selection of a homogeneous study group, etc. The analysis of the literature also highlights solutions such as strong collaboration between emergency medical services (EMS) and hospital care, use of (mobile) health technologies and artificial intelligence, use of standardized protocols and guidelines, etc. Overall, the purpose of this narrative review is to examine the current state of clinical research in prehospital care and identify gaps in knowledge, including the challenges and opportunities for future research.

## 1. Introduction

Prehospital care is a crucial aspect of emergency medicine that involves providing medical assistance to patients before they arrive at a hospital or healthcare facility [1]. According to the World Health Organization (WHO), injuries and illnesses that require emergency care affect millions of people globally, with many of these incidents occurring in low- and middle-income countries [2]. Despite the importance of prehospital care, there are significant disparities in access to this care around the world. In some areas, there may be limited or no access to emergency medical services, while in others, the quality of care may be inadequate. Recently, the American College of Emergency Physicians (ACEP) International Ambassador Country Reports shed light on the varying levels of access to emergency medical services worldwide with certain observations: disparities in access, infrastructure and resource constraints, geographical and financial barriers, lack of international collaboration, etc. [3]. Despite these challenges, efforts are underway to improve access to prehospital care and reduce disparities worldwide. Organizations such as the WHO and the International Federation of Red Cross and Red Crescent Societies are working to improve training and resources for emergency medical services (EMS) personnel and expand access to emergency medical services in underserved areas [4].

Prehospital care is often the first point of contact between a patient and the healthcare system and plays a critical role in reducing mortality and morbidity associated with acute illnesses and injuries. Clinical research in prehospital care is essential to ensure that the care provided to patients in this environment is evidence-based and effective. This type of care is typically delivered by EMS personnel, who are trained to provide a range of treatments and interventions to stabilize patients and prepare them for transport to a hospital [5]. Prehospital care plays a vital role in improving patient outcomes, as early intervention can often mean the difference between life and death.

The use of evidence-based guidelines and protocols can help to improve the quality of care provided in the prehospital environment, reduce the risk of adverse events, and improve patient outcomes [6]. With an acceleration over the past decade, prehospital care has become increasingly sophisticated, with advances in technology and medical treatments allowing EMS personnel to deliver more advanced care in the field. The integration of technology in prehospital care is becoming increasingly pivotal for enhancing patient outcomes. Notably, health technologies such as telemedicine and mobile health tools—encompassing smartphones, tablets, and wearable devices—are driving this progress. These innovations hold the potential to significantly enhance communication between prehospital care providers and healthcare experts [7,8,9]. The advent of mobile technology has empowered EMS personnel to transmit real-time patient data, encompassing crucial metrics such as vital signs and electrocardiograms, to hospitals and healthcare practitioners. This facilitates hospitals in preparing for the patient’s arrival, ensuring the availability of essential resources and medical staff for immediate care. In addition, new medical treatments and procedures are now available in developed countries to EMS personnel in the field, allowing them to provide advanced care that was previously only available in a hospital setting [10]. For example, paramedics can now administer intravenous medications, perform advanced airway management or ultrasonography in life-threatening conditions, utilize new devices for rapid intraosseous access, operate analyzers of cardiac markers or electrolytes in ambulances, and even perform life-saving procedures such as needle decompression of a tension pneumothorax [11,12,13,14]. Overall, technology and medical advancements in prehospital care are transforming the field and improving patient outcomes.

In recent years, there has been growing interest in clinical research in prehospital care. Clinical research in prehospital care is necessary to provide healthcare professionals with evidence-based guidelines and protocols for the treatment of patients in this environment [15]. Indeed, and despite this progress, there are still significant gaps in our understanding of the best approaches to prehospital care [16]. One challenge in conducting clinical research in prehospital care is the limited availability of resources and infrastructure, ethical and regulatory considerations, time constraints, safety concerns, data collection, and the difficulty of selecting a homogeneous study group of patient [17]. Prehospital care providers often work in challenging environments, with limited resources and time constraints. This can make it difficult to conduct high-quality research in this field. Another challenge in conducting clinical research in prehospital care is the need to balance research with the provision of timely and appropriate care to patients. Prehospital care providers must always prioritize the needs of their patients, which can sometimes conflict with the needs of researchers. Despite these challenges, there have been many important advances in clinical research in prehospital care. For example, randomized controlled trials (RCTs) have been used to evaluate the effectiveness of different interventions in the prehospital environment, including advanced airway management, pain management, and the use of mechanical chest compressions in cardiac arrest [18,19,20]. In addition, observational studies have been used to identify risk factors for adverse events in the prehospital environment and to evaluate the effectiveness of prehospital care protocols and guidelines. The use of observational studies has also allowed researchers to identify gaps in the best approaches to prehospital care and to develop hypotheses for future research [21].

For the purposes of this narrative review, we conducted a search in the PubMed electronic database [22]—the most commonly used search platform for medical literature and for scientific literature published in English—up to April 2023; “prehospital care”, “prehospital research”, “emergency medical research”, “prehospital quality”, and “prehospital technology” were used as search terms. Additional references were retrieved from reviewing the references cited in the original articles. All methodological human studies were included in this review (e.g., single-center, multi-center, randomized or not, prospective or retrospective studies, etc.).

Within the scope of this review, several key factors come to the forefront for examination. These include limited resources and infrastructure, ethical and regulatory considerations, time constraints, data collection methods, privacy, and safety concerns, as well as the challenges related to selecting a homogeneous study group. Furthermore, the literature analysis underscores potential solutions to these challenges. These solutions encompass fostering robust collaboration between Emergency Medical Services (EMS) and hospital care, leveraging (mobile) health technologies and artificial intelligence, and adopting standardized protocols and guidelines. In essence, this narrative review’s overarching aim is to assess the current landscape of clinical research in prehospital care while pinpointing areas where knowledge gaps persist. Additionally, it seeks to shed light on the challenges and opportunities that will shape future research endeavors.

## 2. Research in Prehospital Care: State of the Art

Prehospital care is a rapidly evolving field, with ongoing research aiming to improve patient outcomes and optimize emergency medical services [23]. Current clinical research studies in prehospital care are investigating a range of topics, including airway management, hemorrhage control, pain management, and stroke care [24,25]. Clinical research in prehospital care aims to identify the best practices and evidence-based approaches to managing acute illnesses and injuries before the patient is transported to a hospital [6].

In the past, there was little field-focused research in the prehospital setting. However, as EMS systems became more established and technology improved, the need for evidence-based approaches to prehospital care became apparent. One of the earliest examples of clinical research in prehospital care was the development of the Advanced Trauma Life Support (ATLS) program in the 1970s [26,27]. The program is a systematic approach to managing trauma patients in emergency situations. It was developed by the American College of Surgeons (ACS) as a standard of care for the initial assessment and treatment of trauma patients. The ATLS program was extended to prehospital care and has since undergone several updates by promoting standardized care, data collection, quality improvement, and collaboration among healthcare professionals. Table 1 provides some examples of clinical research studies conducted in prehospital care from 1980 to 2020, with a focus on their findings and implications for practice [28,29,30,31,32,33,34,35,36,37,38,39,40,41,42,43,44,45,46]. The selection of factors was based on the available evidence and their practical implication in prehospital research. For the presentation of the findings in Table 1, Table 2 and Table 3, a categorization based on their relation to important research studies, evidence-based practice guidelines, and potential for artificial intelligence was applied. These studies cover a range of topics in prehospital care, including trauma management, airway management, cardiac arrest, and intravenous fluid replacement therapy. Other studies are evaluating the use of tranexamic acid and tourniquets to control hemorrhage in trauma patients, with a focus on prehospital administration and the impact on survival rates. All this research provides important insights into the best practices for prehospital care during the late 20th century.

A new frontier in prehospital care is the enhancement of various aspects, such as airway management, pain control, and stroke care. In the realm of airway management, ongoing research is delving into the utilization of video laryngoscopy, supraglottic airway devices, and neuromuscular blocking agents. These investigations aim to bolster the success rate and safety of endotracheal intubation [47]. Pain management also takes center stage in prehospital care, with active studies examining the efficacy of intranasal fentanyl and ketamine in providing relief to patients experiencing acute pain [48]. Furthermore, the field of stroke care is experiencing substantial growth in research endeavors. Studies are now exploring the deployment of mobile stroke units, equipped with advanced imaging and treatment capabilities, to extend rapid and effective care to individuals exhibiting acute stroke symptoms [49].

Another milestone in the evolution of clinical research in prehospital care came from the United States and was the establishment of the National Emergency Medical Services Information System (NEMSIS) in the early 2000s. NEMSIS is a standardized data collection and reporting system (including patient demographics, clinical outcomes, and interventions) specifically designed for EMS agencies in all the United States. This collaborative effort among federal agencies, EMS stakeholders, and state EMS offices to identify trends in prehospital care and to establish a national standard for collecting and sharing EMS data [50].

In recent years, there has been an increased focus on the use of randomized controlled trials (RCTs) in prehospital care research. RCTs are considered the gold standard for evaluating the effectiveness of medical interventions, and their use in prehospital care has led to significant advancements in the field. For example, a recent RCT compared the use of prehospital epinephrine to placebo in patients with out-of-hospital cardiac arrest, and found that epinephrine improved rates of survival to hospital discharge [51]. The study was conducted as a randomized, double-blind trial involving 8014 patients in 10 different countries. The patients were randomly assigned to receive either epinephrine or a placebo during resuscitation efforts. The primary outcome measure of the study was survival to hospital discharge with a favorable neurologic outcome. Secondary outcomes included return of spontaneous circulation, survival to hospital admission, and adverse events. The study found that the rate of survival to hospital discharge with a favorable neurologic outcome was higher in the group that received epinephrine compared to the placebo group. However, the study also found that the use of epinephrine was associated with a higher rate of severe neurological impairment among survivors. The study highlights the need for further research and improved resuscitation techniques to improve patient outcomes in cardiac arrest situations.

In addition to RCTs, there has also been an increased focus on the use of observational studies in prehospital care research. Observational studies allow researchers to evaluate the effectiveness of interventions in real-world settings and can provide valuable insights into the effectiveness of interventions that may not be feasible to study using RCTs. For example, a recent observational study assessed the effects of prehospital resuscitation with hypertonic solutions on coagulation and fibrinolysis in patients with traumatic hemorrhagic shock. The study included 34 patients who received prehospital resuscitation with hypertonic saline and dextran and the study highlights the potential negative effects of prehospital resuscitation with hypertonic solutions in patients with traumatic hemorrhagic shock, particularly on coagulation and fibrinolysis, and supports the need for further research to determine optimal resuscitation strategies for these patients [52].

## 3. Research in Prehospital Care: Major Challenges

**Figure 1:**
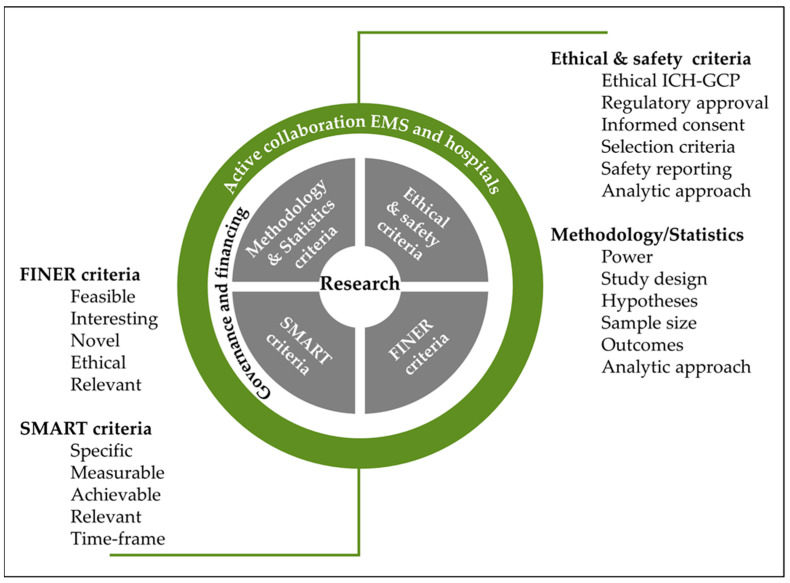
Feasibility criteria needed for the development and success of a clinical research project in prehospital care.

Conducting clinical research in prehospital care presents several unique challenges that can impede the quality and feasibility of studies in this field. From limited resources and logistical constraints to ethical considerations and patient safety concerns, prehospital research requires careful planning and execution to ensure valid and reliable results. Understanding the challenges and opportunities in prehospital clinical research is essential for advancing the field and improving patient care. In this context, Figure 1 shows how the feasibility phase is clearly the most important for any clinical researcher and refers to the necessary limited steps (scientific methodology, people management skills, ethics and regulatory compliance, financial dynamics, participant recruitment, information technology & systems, institutional commitment, how to calculate the sample size and power of the study, fixing the objectives/endpoints, etc.), how all of these are organized, and how they communicate operationally (for activities such as financing, patient recruitment, informed consent process, safety and deviation to protocol reporting, investigational medicinal product administration/destruction, staff training, etc.) to design clinical research (from observational to investigational clinical phases) within the action plan. The first questions that needs to be answered before conducting research in prehospital care can be summed up as:

- “What are the aims of the study?”, which encompasses the SMART (Specific, Measurable, Achievable, Relevant, and Time-frame) criteria (Figure 1) and are linked to methodology/statistics.
- “Why, Where and How should the study be conducted?”, which encompasses the FINER (feasible, interesting, novel, ethical, relevant) criteria (Figure 1) and are linked to ethical/safety criteria.

### 3.1. Limited Resources and Infrastructure

The limited resources available in prehospital care for research pose significant barriers to scientific investigations, which can result in inadequate sample sizes, incomplete data collection, and inconsistent outcomes. These challenges are primarily due to the nature of prehospital care, where medical personnel have to manage emergency situations with limited time, resources, and information [53]. One of the most significant challenges in conducting research in prehospital care is the limited availability of funding. Prehospital care is often underfunded and receives less attention compared to other areas of healthcare. The lack of funding means that there is a limited pool of resources available to support research in this field. As a result, many researchers struggle to access funding to support their investigations, which can lead to underpowered studies with limited generalizability [54]. Emergency medical services personnel are often overworked, and their primary focus is on providing immediate medical care to patients [55]. As a result, it can be challenging to recruit personnel to participate in research studies or to allocate time to collect data. Additionally, prehospital care infrastructure is often decentralized, with services provided by multiple organizations with different protocols and resources [56]. This can make it difficult to standardize data collection and analysis across different regions, leading to inconsistent outcomes. The lack of access to prehospital care data is another significant barrier to conducting research in this field [57]. Prehospital care data are often fragmented and dispersed across multiple agencies, making it difficult to collect, integrate, and analyze the data. There are also issues related to data privacy and security that can limit the sharing of data between agencies and organizations, further complicating the research process [58]. Despite these challenges, there is a growing recognition of the importance of prehospital care research in improving patient outcomes and optimizing the delivery of care.

### 3.2. Ethical Considerations

Conducting clinical research in the prehospital setting can be challenging due to a number of ethical considerations that need to be taken into account [59,60]. These include issues related to informed consent, confidentiality, privacy, and autonomy. In addition, there are unique challenges related to the prehospital setting, such as time constraints, patient acuity, and the potential for emergencies that can further complicate the ethical considerations of conducting research in this setting. One of the main ethical considerations in prehospital research is informed consent. Patients in the prehospital setting are often in a state of distress, and may not be able to provide informed consent for research participation [61]. Additionally, there may be situations where informed consent cannot be obtained in a timely manner due to the patient’s condition or the urgency of the situation [62]. In such cases, alternative methods of obtaining informed consent, such as deferred or waived consent, may need to be considered [63]. Confidentiality and privacy are also important ethical considerations in prehospital research. Patients’ medical information is sensitive and must be protected. Researchers must take steps to ensure that patients’ data are kept confidential and are not shared with unauthorized individuals. Additionally, patients’ right to privacy must be respected, and their personal information should only be collected for research purposes that are clearly defined and explained. Another important ethical consideration in prehospital research is autonomy. Patients have the right to make decisions about their own care, and their autonomy should be respected in the research process as well. Patients should be given the opportunity to decline participation in research, and their decisions should be respected without negative consequences to their care [64]. Overall, ethical considerations in prehospital care for research are essential to ensure that research is conducted in a manner that is respectful, safe, and beneficial to patients. It is important for researchers and EMS providers to be aware of these ethical considerations and to take them into account when designing and conducting research studies in the prehospital setting.

### 3.3. Impact of Time Constraints

Time is of the essence in prehospital care. Emergency medical responders must work quickly and efficiently to provide critical care to patients in emergency situations. Whether it is a heart attack, stroke, or traumatic injury, every second counts in providing life-saving treatment to those in need [65,66]. With time being such a critical factor in prehospital care, emergency medical responders must be able to work under pressure and prioritize their actions to maximize the chances of a positive outcome. In this context, understanding and effectively managing time constraints in prehospital care is essential for saving lives and improving patient outcomes.

Researchers may have a limited amount of time to collect data in prehospital care settings, such as in the case of observing emergency medical responders during real-life situations. They must be able to collect accurate and meaningful data in a short period of time while minimizing the impact on the care provided to patients [67]. Time constraints can arise due to a variety of factors, such as the need to rapidly stabilize and transport patients, the unpredictability of emergency situations, and the limited availability of EMS resources [68]. To maximize the benefits of prehospital research, it is essential to address the time constraints on study conduct, data collection, analysis, and privacy associated with conducting studies in this setting [69].

### 3.4. Safety Concerns

Prehospital care providers often operate in high-stress environments, where they are required to make rapid and accurate decisions to ensure the best possible outcomes for their patients. One of the most significant challenges in conducting research in prehospital care is ensuring the safety of both patients and prehospital care providers.

Due to the urgent nature of prehospital care, prehospital care providers are frequently exposed to hazardous conditions or violent incidents, which can increase the risk of injury or harm. To address these concerns, researchers must take appropriate measures to ensure the safety and well-being of all individuals involved in prehospital care research. This may involve implementing strict protocols to minimize risks and ensuring that all prehospital care providers receive proper training and education on research protocols [70]. These protocols and procedures are designed to ensure that patients receive the highest quality of care while minimizing the potential for medical errors or adverse events. These protocols can include procedures for assessing patient needs, determining the appropriate course of treatment, and transporting patients to the hospital safely [71]. The challenges and risks associated with prehospital care can range from environmental factors such as adverse weather conditions, to patient-specific factors such as the severity of the patient’s condition, the presence of comorbidities, and the patient’s age or decisions [72]. Additionally, healthcare providers must contend with transportation-related risks, such as accidents or equipment malfunctions [73].

A significant challenges is the lack of access to medical resources that are typically available in a hospital setting [74]. This means that prehospital providers must be able to make quick decisions based on the information they have available, often with limited resources at their disposal. This can lead to situations where healthcare providers must rely on their training and experience to provide care in a timely and effective manner. Another challenge in prehospital care is the need for effective communication between healthcare providers [75]. Prehospital providers must be able to communicate effectively with each other, as well as with hospital staff, to ensure that patients receive the care they need. Communication breakdowns can lead to delays in treatment, misdiagnoses, and other adverse events. Training is also an essential component of ensuring safety in prehospital care. Healthcare providers must undergo extensive training to learn how to assess patient needs, provide appropriate care, and respond to emergencies. Ongoing training and continuing education are also critical to ensuring that healthcare providers stay up-to-date with the latest techniques and best practices [76]. Another critical factor is the use of appropriate equipment and technology. Healthcare providers rely on a wide range of tools and equipment to provide care, such as defibrillators, oxygen tanks, and stretchers. Ensuring that this equipment is well maintained and functioning correctly is essential to providing safe and effective care [77].

Healthcare providers must contend with a wide range of challenges and risks when providing care outside of a hospital setting, and safety protocols and procedures are essential to mitigating these risks [78]. Effective communication, ongoing training, and the use of appropriate equipment and technology are all critical components of ensuring patient safety in prehospital care. By prioritizing safety in every aspect of prehospital care, healthcare providers can improve patient outcomes and provide the highest quality of care possible.

### 3.5. Data Collection and Analysis

There are several challenges associated with data collection and analysis in prehospital care for research purposes [79]. These challenges can include difficulties in obtaining informed consent from patients, the need to prioritize patient care over research data collection, and the lack of standardized data collection tools and methods. In addition, prehospital care providers often work in diverse and geographically dispersed settings, which can make it difficult to coordinate data collection efforts and ensure consistency across different study sites. Despite these challenges, there have been significant advances in the field of prehospital care research, with many studies demonstrating the potential benefits of collecting and analyzing prehospital data [80]. By examining prehospital care interventions and outcomes, researchers can identify areas for improvement, evaluate the effectiveness of new treatments, and inform evidence-based practice guidelines. To address the challenges of data collection and analysis in prehospital care research, it is essential to develop standardized data collection tools and methods that can be easily implemented across different study sites [81]. Table 2 provides some historical references of evidence-based practice guidelines and standardized protocols for prehospital care, with a focus on their findings and implications for practice [82,83,84,85,86,87,88,89,90,91,92,93,94].

**Table 2:** Examples (in chronological order) of evidence-based practice guidelines and standardized protocols for prehospital care.

Findings	Target Population	Study Design
Rottman et al. (1997) compared on-scene time, appropriateness of therapy, and accuracy of paramedic clinical assessments when prehospital care was provided with the use of on-line medical control (OLMC) by EMS-certified nurses from a single base station or by paramedics using chief complaint-based protocols. The use of protocols resulted in small improvements in both on-scene time and the appropriateness of therapeutic decisions, without a change in agreement between paramedic and physician [82].	EMS call center	Prospective cohort
Holstein et al. (2003) found that training of the emergency team is an effective and efficient intervention to improve quality of treatment and prognosis outcome for patients with type 1 diabetic emergencies [83].	Diabetic patients	Prospective population-based study
Watts et al. (2004) found that providers who were able to learn and implement the Brain Trauma Foundation (BTF) Guidelines and outcomes in traumatic brain injury patients were significantly improved [84].	Traumatic brain injury patients	Prospective observational study
Combes et al. (2006) determined the rate of difficult intubations and the factors associated with prehospital difficult airways when a standard protocol for sedation and intubation was applied [85].	Tracheal intubation patients	Observational et prospective study
Sasson et al. (2009) discussed the operational issues within local EMS systems that may serve as barriers or facilitators to full acceptance of national guidelines for prehospital termination of resuscitation in appropriate circumstances [86].	Termination of resuscitation	Qualitative and focus groups study
Atary et al. (2010) showed that a standardized regional acute myocardial infarction treatment protocol achieved optimal and uniformly distributed pre-hospital performance in the region ‘Hollands-Midden’, resulting in minimal time delays regardless of area of residence [87].	Myocardial injuries patients	Standardized pre-hospital care guidelines applied in practice
Rognas et al. (2013) reported a prospective quality control study of the effect on pre-hospital critical care anesthesiologists’ behavior of implementing a standard operating procedure for pre-hospital controlled ventilation [88].	Airway management patients	Prospective registry
Brandler et al. (2015) found that EMS care providers missed more than a third of stroke cases. Seizures and other atypical presentations contribute significantly to stroke misdiagnosis [89].	Prehospital stroke identification methods	Retrospective report
Osborne et al. (2015) summarized the United Kingdom (UK) Ambulance Service guidelines for the management of seizures and explored the extent to which these guidelines are evidence-based [90].	Management of seizures	Guidelines report
Kerner et al. (2017) evaluated how the use of checklists for prehospital emergency care may help to improve adherence to treatment guidelines [91].	Checklists in prehospital emergency care	Standard operating procedures study
Lenssen et al. (2017) suggested that routine, remote, physician-based, telemedically-delegated (opioid-based) analgesia in trauma and non-trauma emergencies, as applied by paramedics, shows comparable efficacy to analgesia administered by on-scene prehospital EMS physicians [92].	Analgesia management patients	Retrospective observational study
Pride et al. (2017) discussed the importance of prehospital care delivery and triage in cases of stroke with emergent large vessel occlusion (ELVO) [93].	Stroke patients	Guidelines report
Rodríguez et al. (2020) found that the use of early warning scores can help the EMS to differentiate traumatic brain injury patients with a high risk of deterioration [94].	Traumatic brain injury patients	Prospective cohort

Finally, close collaboration between prehospital care providers, researchers, and other stakeholders is essential to ensure that data are collected and analyzed in a way that maximizes their value and potential impact on patient care [95]. Overall, data collection and analysis are critical components of prehospital care research, and the challenges associated with these activities must be carefully considered and addressed to advance the field and improve patient outcomes.

### 3.6. Selection of a Homogeneous Study Group

One of the crucial challenges encountered in prehospital care research is the difficulty of selecting a homogeneous study group. The unique nature of prehospital care, with its diverse patient population and varying emergency scenarios, presents researchers with numerous complexities when it comes to forming a cohesive and homogeneous study group. Paramedics and emergency medical service providers encounter an extensive range of medical conditions, injuries, and socioeconomic backgrounds among patients they treat. From traumatic injuries to cardiac arrests, respiratory distress to neurological emergencies, the diversity of cases encountered in prehospital care is vast and constantly evolving. This difficulty goes beyond its impact on the validity and generalizability of findings. It also has practical implications for the translation of research outcomes into clinical practice. Healthcare providers rely on evidence-based guidelines derived from rigorous research to inform their decision-making process in emergency situations. If the study groups lack homogeneity, the applicability and relevance of the research findings may be compromised, impeding the development of effective interventions and guidelines for prehospital care. Hence the need to better identify emergency phenotypes [96,97].

## 4. Discussion

Overall, clinical research in prehospital care is an essential component of improving the quality of care provided to patients in this environment. Despite the challenges, researchers in this field have made significant progress in identifying effective interventions and improving patient outcomes. For example, studies can help to identify best practices for responding to emergencies and treating specific conditions, as well as to develop new technologies and interventions for use in prehospital care. So, continued research and innovation will be critical to ensuring that prehospital care providers have access to evidence-based guidelines and protocols for the treatment of patients in this critical setting.

**Figure 2:**
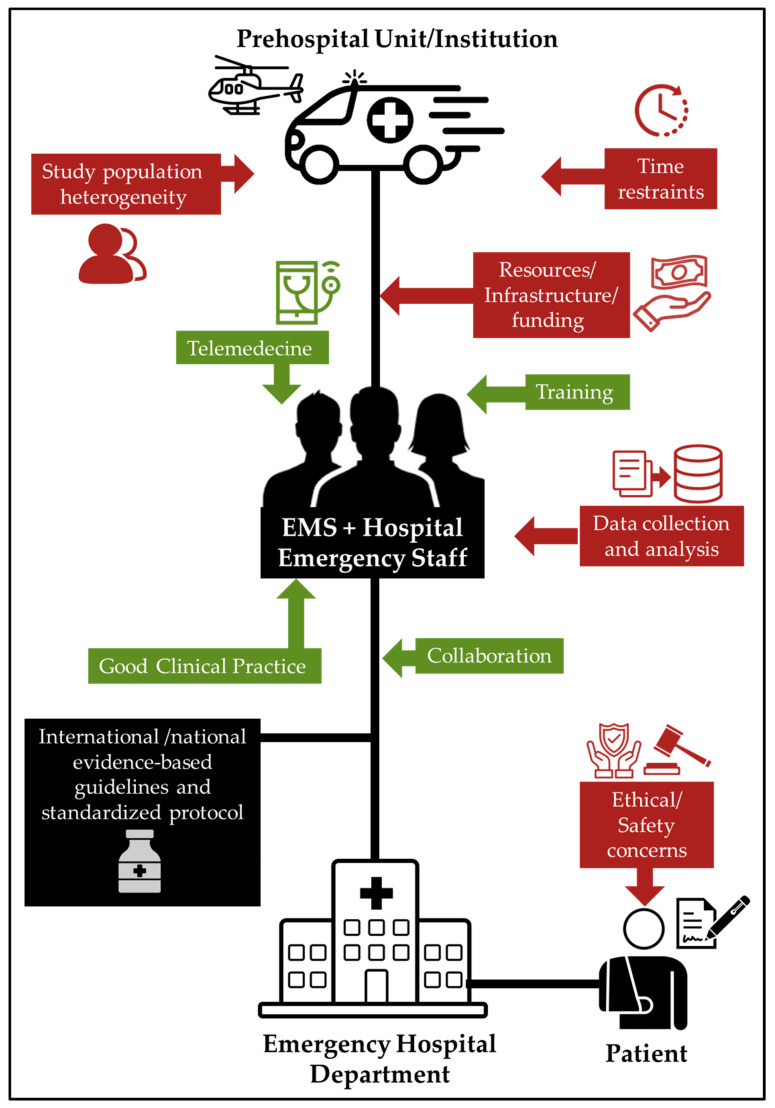
EMS and hospital care research activities: balance between challenges (red) and opportunities (green).

To address the challenges facing prehospital clinical research, researchers must carefully plan and execute their studies, taking into account the unique constraints and considerations of prehospital care. This may involve working closely with EMS agencies and other healthcare providers to develop study protocols and ensure that studies are conducted in a safe and ethical manner. Additionally, researchers may need to leverage new technologies and data sources to collect and analyze data from prehospital care environments (Figure 2).

### 4.1. What Are the Solutions to Implement?

There are several solutions that can be implemented to improve clinical research in prehospital care (Figure 2):

Collaboration: Collaboration between prehospital care providers, hospitals, and research institutions can improve the quality of research in prehospital care. This collaboration can lead to better study design, more robust data collection, and stronger analysis.

Technology: Technology can be leveraged to improve data collection and analysis. For example, the use of electronic health records (EHRs) can help standardize data collection and make it easier to share data between prehospital care providers and researchers. Additionally, the use of mobile apps and wearables can provide real-time data that can be used for research purposes.

Training: Prehospital care providers should receive training on research methods and data collection to ensure they are collecting data in a standardized and accurate manner. This training can also help providers understand the importance of research and the impact it can have on patient care.

Funding: Increased funding for prehospital care research can help support larger, more comprehensive studies. This funding can also be used to develop new technologies and research methods to improve data collection and analysis.

Ethics committees: Ethical considerations must be addressed when conducting research in prehospital care. Establishing ethics committees that review research proposals and ensure that patient privacy and safety are maintained can help improve the quality and trustworthiness of research in prehospital care.

Public awareness: Greater public awareness of the importance of prehospital care research can help increase funding and support for this area of study. It can also help improve patient participation in research studies and increase the overall impact of the research.

In practice, collaboration, technology, training, funding, ethics committees, and public awareness can all contribute to improving clinical research in prehospital care. By addressing the challenges and implementing solutions, we can improve our understanding of prehospital care and ultimately improve patient outcomes.

### 4.2. What Are the Future Opportunities and Perspectives?

As technology advances, new treatments and interventions emerge, and the landscape of prehospital care is continually evolving. Despite the challenges discussed earlier, there are also several opportunities and perspectives for improving clinical research in prehospital care.

First, the use of telemedicine and remote monitoring has the potential to improve data collection and analysis in prehospital care research. Telemedicine enables real-time communication between emergency medical services (EMS) providers and remote medical professionals, allowing for the exchange of vital patient information and coordination of care. Remote monitoring technologies can also collect data on patients’ vital signs and other metrics in real time, providing valuable insights into patient outcomes and treatment effectiveness. The advent of telemedicine has also played a role in the evolution of clinical research in prehospital care. Telemedicine allows EMS providers to consult with physicians and other healthcare providers in real-time, providing access to expert advice and improving patient outcomes [98,99]. One example of the use of telemedicine in prehospital care is the Stroke Prehospital Assessment and Treatment program, which allows EMS providers to transmit brain imaging and other data to stroke specialists in real time, improving the speed and accuracy of diagnosis and treatment. The study from Katz et al. outlines the development and validation of the Cincinnati Prehospital Stroke Severity Scale, which is a key component of this program [100]. Besides that, there are also other connected technologies that allow for improved data collection and information distribution (movement tracking devices, video and audio recording, running simulations, IT infrastructure, etc.) [101,102,103,104,105,106,107,108,109,110].

Second, the use of simulation-based training for EMS providers can improve the quality of care delivered in prehospital settings. Simulation-based training allows EMS providers to practice responding to a wide range of emergency scenarios in a safe and controlled environment, improving their skills and confidence in delivering effective care. Moreover, simulation-based training can help researchers evaluate the effectiveness of new interventions and treatments in prehospital care [111].

Third, the development and use of standardized protocols and guidelines for prehospital care can improve the quality and consistency of care delivered by EMS providers. Standardized protocols can also facilitate the evaluation of new treatments and interventions by providing a consistent framework for comparing outcomes across different settings and populations [112].

Finally, with the advent of artificial intelligence (AI) technologies, there is great potential to enhance prehospital care by providing faster and more accurate assessments of patient conditions, and enabling more efficient allocation of resources. Prehospital AI refers to the use of machine learning algorithms and other AI techniques to improve emergency medical care before the patient arrives at the hospital. One area where prehospital AI is being explored is in the use of predictive models to help identify patients who are at risk of deteriorating rapidly or experiencing a cardiac arrest. By analyzing patient data such as vital signs, medical history, and demographic information, these models can provide early warnings to EMS personnel, allowing them to intervene quickly and potentially prevent a critical event from occurring. Other applications of prehospital AI include automated triage, diagnosis support, and resource allocation optimization [113,114,115,116,117,118,119,120,121,122]. Table 3 provides a comprehensive overview of clinical research studies conducted in prehospital care using AI, with a focus on their findings and implications for practice [123,124,125,126,127,128,129].

**Table 3:** Examples of studies (in chronological order) that demonstrate the potential for artificial intelligence to enhance prehospital care.

Findings	Target Population	Study Design
Liu et al. (2014) highlighted the potential for machine learning algorithms to improve the accuracy of predicting the need for life-saving interventions in trauma patients, enabling faster and more appropriate treatment for these patients. The hybrid system developed in this study may also serve as a model for integrating machine learning algorithms into clinical decision-making processes [123].	Analgesia management patients	Retrospective and prospective cohort study
Desautels et al. (2016) highlighted the potential for machine learning models to improve sepsis prediction in the ICU using minimal EHR data, which may be particularly useful in resource-limited settings. However, the study also acknowledges the limitations of using retrospective data and the need for prospective validation of the models in clinical practice [124].	Sepsis prediction	Retrospective study
Cheng et al. (2021) highlighted the potential for deep learning algorithms to assist sonographers in the detection of abdominal free fluid in Morison’s pouch during sonography in trauma, potentially enabling faster and more accurate diagnosis of abdominal trauma. However, the study also acknowledges the limitations of using retrospective data and the need for prospective validation in clinical practice [125].	Abdominal trauma patients	Observational study
Fontanellaz et al. (2021) highlighted the potential for deep learning algorithms to assist radiologists in the detection of COVID-19 using chest radiographs, potentially enabling faster and more accurate diagnosis of the disease. However, the study also acknowledges the limitations of using retrospective data and the need for prospective validation of the deep learning diagnostic support system in clinical practice [126].	COVID-19 patients	Retrospective study
Uchida et al. (2021) demonstrated the feasibility and effectiveness of using machine learning algorithms as a diagnostic support tool in the prediction of stroke probability and type at the prehospital stage, potentially leading to improved stroke care and patient outcomes [127].	Stroke-management patients	Retrospective and prospective cohort study
Shahi et al. (2021) highlighted the potential for deep learning algorithms to improve decision-making in pediatric blunt solid organ injury, enabling faster and more accurate predictions of the need for massive transfusion, need for operative management, and mortality risk. The use of deep learning algorithms in trauma care may also reduce healthcare costs and improve patient outcomes [128].	Pediatric blunt solid organ injury	Retrospective study
Chen et al. (2022) highlighted the potential for AI-assisted systems to improve prehospital care by enabling faster and more accurate detection of ST-elevation myocardial infarction, which is crucial for timely intervention and improved patient outcomes. The use of a mini-12-lead ECG device also makes the system more accessible for use in resource-limited settings [129].	Myocardial injury patients	Retrospective study

## 5. Limitations

The approaches cited in this article have the potential to improve patient outcomes and advance the field of prehospital care. However, as a narrative review, there are some limitations. The first is that rather than focusing on recent research in the last five years, this review has included historical and influential scientific studies that may no longer be relevant in the current setting. It is a limitation that the authors did not begin with a research question when conducting the review; therefore, there was no guide as to what information would be significant and what might be circumstantial. It is a limitation that the authors did not conduct any pooled analyses of the data from the studies summarized. Additionally, searches were only conducted in one database.

## 6. Conclusions

In conclusion, clinical research in prehospital care is essential for improving patient outcomes and developing evidence-based best practices. Overall, the evolution of clinical research in prehospital care has led to significant advancements in the field, improving outcomes for patients with acute medical emergencies. However, the field of prehospital care presents several challenges to clinical research, including limited resources, ethical considerations, time constraints, safety concerns, and data collection and analysis. Fortunately, there are also several opportunities and perspectives for improving clinical research in this field, including the use of telemedicine and remote monitoring, simulation-based training, standardized protocols and guidelines, and collaborations between EMS providers, hospitals, and academic institutions.

## Figures and Tables

**Table 1:** Examples of research studies (in chronological order) from a given prehospital “potential killer” conducted in pre-hospital care.

Findings	Target Population	Study Design
Cummins et al. (1985) examined the records of 1297 people with witnessed out-of-hospital cardiac arrest caused by heart disease and treated by both emergency medical technicians (EMTs) and paramedics, to determine early cardiopulmonary resuscitation (CPR) improved survival [28].	Cardiac arrest patients	Observational
Bickell et al. (1994) found that prehospital intravenous fluid replacement therapy improved survival in hypotensive trauma patients, highlighting the importance of aggressive fluid resuscitation in this population [29].	Hypotensive trauma patients	Prospective cohort
Sampalis et al. (1999) showed that tertiary trauma centers and reduced prehospital times are the essential components of an efficient trauma care system [30].	Traumatic injury patients	Prospective cohort
Gausche et al. (2000) found that out-of-hospital pediatric endotracheal intubation did not improve survival or neurological outcomes compared to bag–mask ventilation, highlighting the challenges of airway management in pediatric patients [31].	Endotracheal intubation in pediatric patients	Controlled clinical trial
Davis et al. (2005) found that prehospital oxygen therapy did not improve mortality in severe traumatic brain injury patients, challenging the previous standard of care [32].	Brain injury patients	Retrospective registry
Richard et al. (2006) shed light on the management and outcomes of pediatric patients transported by EMS in a Canadian prehospital system. Through an analysis of a prospective cohort, the research provides valuable insights into the characteristics, interventions, and outcomes of pediatric patients in the prehospital setting [33].	Pediatric emergency medicine	Prospective cohort
Ortolani et al. (2007) demonstrated the significant benefit of prehospital triage in identifying patients with cardiogenic shock complicating ST-elevation myocardial infarction (STEMI) who would benefit from primary percutaneous coronary intervention (PCI). The results indicate that patients who received prehospital triage had a significantly lower mortality rate compared to those who did not undergo prehospital triage. Furthermore, the study reveals that prehospital triage was associated with a higher likelihood of achieving optimal revascularization [34].	Prehospital cardiogenic shock	Prospective registry
Kragh et al. (2009) evaluated the use of tourniquets in trauma patients in war areas. The study found that tourniquet use when shock was absent was strongly associated with saved lives, and prehospital use was also strongly associated with life-saving outcomes [35].	Traumatic injury patients	Prospective survey
Nassif et al. (2009) found that prehospital protocol change for asthmatic children is associated with shorter total hospital and total care times. This protocol change was also associated with decreased hospitalization rates and less need for critical care in those hospitalized. Further study is necessary to determine if other factors also contributed. [36].	Children with minor head trauma	Prospective cohort
Dracup et al. (2009) demonstrated the effectiveness of a targeted educational intervention in reducing prehospital delay to treatment in acute coronary syndrome (ACS) patients. The study emphasizes the importance of patient education and empowerment in promoting timely medical care-seeking behavior. The findings suggest that interventions aimed at improving symptom recognition, knowledge of ACS symptoms, and overcoming barriers can contribute to better outcomes for ACS patients by facilitating early access to appropriate treatments [37].	Acute coronary syndrome patients	Randomized clinical trial
Bergs et al. (2010) demonstrated the feasibility and reliability of prehospital stroke scales in the Belgian prehospital setting. The results indicate that EMS personnel were able to effectively administer and interpret the stroke scales, leading to accurate identification and triage of potential stroke patients. The study also reveals a high level of inter-rater reliability among EMS providers in using the stroke scales [38].	Stroke patients	Prospective cohort
Monsieurs et al. (2012) showed an association between higher compression rates and lower compression depths. Avoiding excessive compression rates may lead to more compressions of sufficient depth [39].	Myocardial infarction patients	Observational
Meretoja et al. (2012) found that the implementation of a prehospital stroke protocol by emergency medical services improved stroke outcomes, emphasizing the importance of early recognition and treatment of stroke symptoms [40].	Stroke patients	Observational
Brown et al. (2015) found that prehospital blood product transfusion improved mortality and functional outcomes in trauma patients during medical evacuation, highlighting the potential benefits of this intervention [41].	Traumatic injury patients	Retrospective cohort
Lockey et al. (2015) found that prehospital advanced life support improved outcomes for major trauma patients, highlighting the importance of early and effective interventions in this population [42].	Major trauma patients	Prospective observational
Crewdson et al. (2017) conducted a systematic review and meta-analysis and found that prehospital rapid sequence intubation was associated with improved outcomes for trauma patients, highlighting the importance of effective airway management in this population [43].	Injury patients	Systematic review and meta-analysis
Wang et al. (2018) compared the effectiveness of two methods of airway management in adults with out-of-hospital cardiac arrest: laryngeal tube (LT) and endotracheal intubation (ETI). Based on these findings, the authors concluded that initial LT insertion may be considered as an alternative to ETI for airway management in adults with out-of-hospital cardiac arrest [44].	Cardiac arrest patients	Randomized clinical trial
Guyette et al. (2021) found that prehospital administration of tranexamic acid after injury did not result in a higher incidence of thrombotic complications or adverse events. Tranexamic acid given to injured patients at risk for hemorrhage in the prehospital setting is safe and associated with survival benefit in specific subgroups of patients [45].	Trauma patients with hypovolemic shock	Randomized clinical trial
Scquizzato et al. (2023) demonstrated that adults with acute respiratory failure treated in the prehospital setting with noninvasive ventilation had a lower risk of intubation than those managed with standard oxygen therapy, with similar risk of death, intensive care admission, and length of hospital stay. [46].	Prehospital respiratory failure	Retrospective cohort
